# WAVE: Wall-Aligned Vector Embedding for Self-Supervised Learning of Electrocardiograms

**DOI:** 10.3390/bioengineering13070733

**Published:** 2026-06-24

**Authors:** Shurong Pan, Wenhan Liu, Qingyuan Wu, Cong Wang, Zhaohui Yuan

**Affiliations:** School of Information and Software Engineering, East China Jiaotong University, Nanchang 330013, China; 3676@ecjtu.edu.cn (S.P.); 2025068085405033@ecjtu.edu.cn (Q.W.); 2025068083500017@ecjtu.edu.cn (C.W.); yuanzh@whu.edu.cn (Z.Y.)

**Keywords:** electrocardiogram, self-supervised learning, deep learning, biosignal processing

## Abstract

Deep learning has achieved remarkable progress in electrocardiogram (ECG) analysis, but its heavy dependence on labeled data greatly increases annotation cost. This work proposes wall-aligned vector embedding (WAVE), a self-supervised learning framework that effectively extracts prior knowledge from unlabeled ECGs to reduce reliance on labels. WAVE fully leverages the diversity, synergy, and lead correlation of multi-lead ECGs by explicitly incorporating the correspondence between ECG leads and cardiac walls. Specifically, a multi-branch network captures lead-wise diversity; wall-wise synergy is modeled by concatenating leads from the same wall and projecting them via shared projection; and a dual alignment task is designed to learn correlations both within and across cardiac walls. Experimental results demonstrate that WAVE consistently surpasses all baselines under various evaluation settings, and maintains strong performance even when only a small fraction of labeled ECGs is available. Furthermore, components such as dual alignment, shared projection, wall-based concatenation, and mean target embedding are empirically verified to significantly enhance pretraining quality. In summary, WAVE learns highly informative ECG representations from unlabeled data, enabling low-cost and label-efficient ECG analysis for real-world cardiovascular diagnostics.

## 1. Introduction

Electrocardiogram (ECG) is a commonly used diagnostic tool for cardiovascular diseases (CVDs). It is characterized by its noninvasive nature and low cost. The standard ECG consists of 12 leads (I, II, III, V1∼V6), which capture the cardiac electrical activity from different spatial perspectives [1]. Cardiologists assess the occurrence of specific conditions by analyzing distinctive alterations in ECG waveforms [1]. However, large scale of ECG data imposes a substantial burden on manual interpretation. In recent years, deep learning has been applied to ECG analysis [2]. These methods enable automated diagnosis of CVDs. These models alleviate the workload of cardiologists and demonstrate considerable potential. Nevertheless, training deep learning models requires large-scale labeled ECG data. The labeling process still relies heavily on expert knowledge, resulting in high costs. Thus, this reliance constitutes a major obstacle to the further advancement of deep learning in ECG analysis. In contrast, unlabeled ECG data can be obtained at low cost, as its collection requires minimal clinical intervention. Leveraging unlabeled ECGs to enhance model training is therefore a promising direction for automated ECG analysis.

Self-supervised learning (SSL) has demonstrated strong capability in leveraging unlabeled data. SSL develops pretext tasks for pretraining on unlabeled datasets. The pretrained models are then transferred to downstream tasks. As pretraining learns prior knowledge from unlabeled data, downstream tasks can achieve competitive performance with only a small amount of labeled samples. This reduces annotation burden. Representative SSL methods include a simple framework for contrastive learning of visual representations (SimCLR) [3], momentum contrast (MoCo) [4], bidirectional encoder representation from image Transformers (BEiT) [5], and masked autoencoder (MAE) [6], among others. These methods have shown promising results across various domains such as computer vision and natural language processing [7].

Specifically, recent studies have adapted these representative methods for SSL in ECG analysis. As reported in [8], SimCLR and BYOL are applied to 12-lead ECG classification. In [9], MoCo is introduced for ECG analysis, and its applicability to wearable devices is also validated. Furthermore, ECG signals are transformed into two-dimensional images. This enables evaluation of BEiT [10]. These studies primarily focus on improving data transformation strategies to make representative SSL methods compatible with ECG signals, without introducing substantial modifications to the SSL mechanisms themselves. In addition, studies in [11,12,13] modify SSL frameworks based on the characteristics of multi-lead ECG, where feature optimization is conducted for each individual lead. However, these studies still fail to sufficiently capture cross-lead feature synergies (as elaborated in Section 3). Overall, these limitations may hinder effective ECG feature learning. They may also restrict performance improvements.

To address the aforementioned limitations, this paper proposes wall-aligned vector embedding (WAVE), a novel SSL method for ECG analysis. WAVE enhances the core SSL mechanism rather than focusing on data transformation strategies. It also considers the wall-wise synergy among lead features by aligning representations across cardiac walls, leveraging the correspondence between leads and the walls. Overall, the contributions of WAVE can be summarized as follows:

- WAVE constructs embeddings for cardiac walls based on a multiple branch network (MBN) and shared projection. It balances lead-wise diversity and wall-wise synergy of features.
- WAVE integrates embedding alignment within each wall and across complementary walls to yield a dual alignment task. It enhances the capacity to capture informative ECG representations.
- WAVE enables models to achieve competitive performance on downstream tasks with only a small amount of labeled ECG data, reducing reliance on manual labeling. Moreover, it consistently outperforms existing methods across different evaluation paradigms.

The remainder of this paper is organized as follows. Section 2 introduces related work, Section 3 details the principles of WAVE, and Section 4 reports the experimental results. Discussions are provided in Section 5, and Section 6 concludes the paper.

## 2. Related Work

### 2.1. Deep Learning for ECG

Essentially, diagnosing CVDs from ECGs can be regarded as an ECG classification task. Deep learning models take ECGs as input and output their corresponding classes (i.e., a specific CVD or normal condition). Typical deep learning architectures such as convolutional neural networks (CNNs), recurrent neural networks (RNNs), and Transformers have all been applied to ECG classification [2]. For instance, Kiranyaz et al. employed a 1D CNN for real-time classification of single-lead ECGs [14]; Hou et al. utilized long short-term memory (LSTM), a variant of RNN, to analyze ECGs and achieved four-class arrhythmia classification [15]; Ji et al. leveraged Transformers to classify ECGs involving multiple types of arrhythmias [16]. Furthermore, many studies have improved deep learning models to adapt to ECG characteristics. Such improvements focus on either micro-level modules or macro-level architectures. Several representative examples are presented below.

For micro-level modules, Chen et al. introduced omni-scale and cross-fusion modules into CNNs, enhancing the model’s sensitivity to both multi-scale and global ECG features [17]. Mantravadi et al. integrated 1D convolution, LSTM, and involution layers to effectively capture both short-range and long-range dependencies in ECGs [18]. Guhdar et al. combined 1D CNNs with a specialized attention mechanism to improve the model’s generalization across different ECG classification tasks [19]. For macro-level architectures, parallel-branch designs are commonly adopted, with each branch dedicated to extracting different types of ECG features. For example, Qi et al. proposed a dual-branch network to simultaneously extract lead-separation and lead-combination features [20], while Jiang et al. designed a dual-branch network to jointly learn limb-lead and precordial-lead features [21]. Moreover, MBNs (mentioned in Section 1) that allocate dedicated branches to each lead have been widely adopted in ECG classification, as demonstrated in [22,23,24]. This design captures both the lead-wise diversity and the integrity of different leads, achieving a balance between lightweight design and strong performance.

Overall, deep learning has made substantial progress in ECG classification, even surpassing human experts in certain scenarios [25]. Nevertheless, these models require large-scale labeled ECG datasets for training, which imposes a significant labeling cost. This reliance on labeled data has become a key bottleneck hindering the broader development of deep learning in ECG analysis.

### 2.2. Self-Supervised Learning for ECG

SSL is an important approach to alleviating the aforementioned dependency. It pretrains models through pretext tasks to acquire prior knowledge. The pretrained models can then be transferred to downstream tasks (e.g., classification), achieving promising performance with only limited labeled data. SimCLR and MoCo are representative SSL methods that pretrain models via contrastive learning, employing joint-embedding architectures and constructing positive–negative sample pairs [3,4]. Bootstrap your own latent (BYOL) and simple Siamese (SimSiam) are variants of contrastive learning that adopt asymmetric architectures to remove the reliance on negative pairs, while still yielding superior performance [26,27]. Barlow twins (BT) and variance-invariance-covariance regularization (VICReg), on the other hand, design pretext tasks from the perspective of reducing feature redundancy. They require neither asymmetric structures nor negative pairs, but at the cost of higher computational overhead [28,29]. In contrast, generative methods such as BEiT and MAE pretrain models via masked image modeling (MIM), without relying on joint-embedding architectures or sample pairs [5,6]. However, these generative methods often underperform contrastive learning in classification tasks [30]. Based on these representative SSL methods, numerous studies have extended their ideas to ECG analysis, as illustrated below.

For contrastive learning, Mehari et al. introduced new ECG-specific transformations, including random resize crop-time out (RRC-TO) and physiological noise (PN), to construct augmented views. The effectiveness is evaluated within contrastive frameworks such as SimCLR and BYOL [8]. Lai et al. proposed four novel ECG transformations and their combinations [9]. Pretraining is conducted with MoCo. Liu et al. leveraged ECG signals from the same individual at different times as positive pairs and employed BYOL for pretraining [31]. For the generative learning, Vaid et al. transformed 12-lead ECGs into images and performed pretraining with BEiT [10]. Na et al. proposed spatio-temporal patchifying and designed a lead-shared encoder–decoder architecture for MAE [32]. Wei et al. simultaneously exploited temporal and frequency-domain representations of ECGs [33]. This is used to develop a bimodal MAE along with its corresponding pretext tasks. Although these methods achieve promising performance, most of them primarily focus on ECG-specific data transformations (e.g., RRC-TO) and representation strategies (e.g., particular patchifying schemes or time–frequency representations), rather than refining the pretraining mechanisms based on ECG characteristics. Liu et al. analyzed multi-lead ECG characteristics and proposed dense lead contrast (DLC) [11], bootstrap each lead’s latent (BELL) [12], and lead correlation & decorrelation (LCD) [13] for SSL in ECG analysis. However, these methods mostly design pretext tasks based on lead-to-lead relationships. They overlook feature synergy across different leads.

Although the above studies have demonstrated the feasibility of SSL in ECG analysis, several limitations still remain. This work addresses these limitations by proposing novel methods to further enhance ECG feature representation learning.

## 3. Method

### 3.1. Motivation

**Figure 1:**
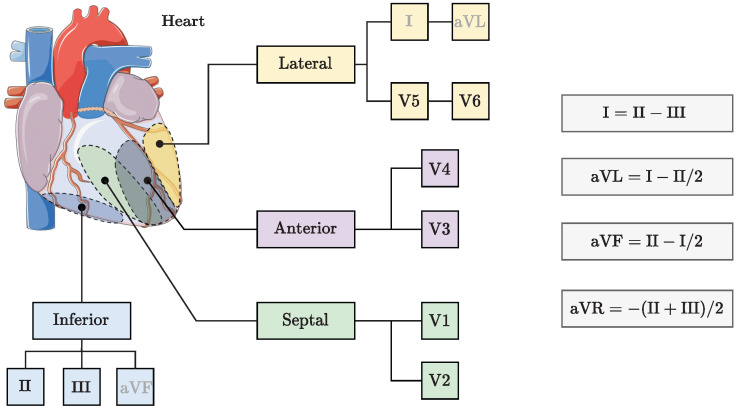
Illustration of the correspondence between cardiac walls and ECG leads [1]. The derivations among limb leads are also shown. Lead names in gray indicate redundancy.

Different ECG leads correspond to distinct spatial perspectives of the heart, and therefore exhibit diverse feature characteristics. Meanwhile, each lead is associated with specific anatomical regions (cardiac walls) of the heart [1], as illustrated in Figure 1. It can be observed that multiple leads may correspond to the same cardiac wall. While pathological conditions (such as diffuse ischemia or multi-vessel myocardial infarction) can cause abnormalities to span across multiple walls, this classic four-quadrant division (inferior, septal, anterior, and lateral walls) serves as a reliable structural anchor. It ensures that leads sharing a dominant spatial perspective are clustered together. These clusters jointly represent the regional physiological state of the heart. Based on these properties, the core design principles of WAVE are summarized as follows:

- Diversity → MBN: The MBN assigns an individual feature extractor to each lead, allowing the distinctiveness of lead-specific features to be preserved.
- Synergy → shared projection: Leads corresponding to the same wall share a projector to form a unified embedding, which facilitates the exploration of feature synergy.
- Correlation → alignment task: Embedding alignment within and across cardiac walls improves the modeling of intra-wall and inter-wall correlations.

Additionally, redundancy exists among the limb leads (I, II, III, aVR, aVL, aVF). Given two limb leads, the remaining six can be algebraically derived. As shown in Figure 1, leads I, aVR, aVL, and aVF can be computed from leads II and III. For manual human interpretation, the standard 12-lead configuration has superior clinical applicability. It presents the heart’s electrical activity from a comprehensive and intuitive geometric framework, which facilitates visual pattern recognition by cardiologists. However, the operational mechanism of deep learning models differs from human vision. Due to the powerful fitting and optimization capabilities of deep neural networks, introducing linearly dependent and redundant channels (such as leads I, aVR, aVL, and aVF, which can be directly and algebraically derived from leads II and III) may lead to overfitting and provide limited benefit for improving generalization. Considering the characteristics of MBN, the 8-lead scheme requires fewer parallel encoders compared to a full 12-lead baseline. This significantly scales down the total parameter footprint and overall computational complexity. This structural compression makes the 8-lead configuration suitable for real-world deployment. Therefore, in this study, only eight leads (II, III, V1∼V6) are retained to reduce redundancy.

### 3.2. Synergistic Effect of Shared Projection

Previous studies have shown that MBN can promote lead-wise diversity [22,23,24], while alignment tasks facilitate the modeling of feature correlations [34]. This subsection further provides a qualitative justification that shared projection facilitates the discovery of wall-wise synergy. Given two feature vectors $h^{\left(1\right)},h^{\left(2\right)}\in R^{d}$ output by two branches, and a target vector $y\in R^{m}$, $h^{\left(1\right)}$ and $h^{\left(2\right)}$ are first concatenated into a vector $h=\left[h^{\left(1\right)};h^{\left(2\right)}\right]$. Without loss of generality, assume that only a single shared projection is applied:

(1) $$\hat{y}=Wh+b=W\left[\begin{array}{c} h^{\left(1\right)}\\ h^{\left(2\right)} \end{array}\right]+b,W\in R^{m\times 2d},b\in R^{m}$$

where W is the shared projection matrix, and b is the bias term. The loss function is defined as mean squared error (MSE):

(2) $$\begin{array}{cc} L & ={∥\hat{y}-y∥}_{2}^{2}={∥W\left[\begin{array}{c} h^{\left(1\right)}\\ h^{\left(2\right)} \end{array}\right]+b-y∥}_{2}^{2}={∥\left[W^{\left(1\right)},W^{\left(2\right)}\right]\left[\begin{array}{c} h^{\left(1\right)}\\ h^{\left(2\right)} \end{array}\right]+b-y∥}_{2}^{2}\\ \; & ={∥W^{\left(1\right)}h^{\left(1\right)}+W^{\left(2\right)}h^{\left(2\right)}+b-y∥}_{2}^{2},W^{\left(1\right)},W^{\left(2\right)}\in R^{m\times d} \end{array}$$

where $W^{\left(1\right)}$ and $W^{\left(2\right)}$ are two submatrices of W. During model training, it is necessary to compute the partial derivatives of the loss L with respect to the features $h^{\left(1\right)}$ and $h^{\left(2\right)}$. Define the error vector $e=W^{\left(1\right)}h^{\left(1\right)}+W^{\left(2\right)}h^{\left(2\right)}+b-y$, then the above partial derivatives can be written as:

(3) $$\left\{\begin{array}{c} \frac{\partial L}{\partial h^{\left(1\right)}}={\left(W^{\left(1\right)}\right)}^{⊤}\left(W^{\left(1\right)}h^{\left(1\right)}+W^{\left(2\right)}h^{\left(2\right)}+b-y\right)={\left(W^{\left(1\right)}\right)}^{⊤}e\\ \frac{\partial L}{\partial h^{\left(2\right)}}={\left(W^{\left(2\right)}\right)}^{⊤}\left(W^{\left(1\right)}h^{\left(1\right)}+W^{\left(2\right)}h^{\left(2\right)}+b-y\right)={\left(W^{\left(2\right)}\right)}^{⊤}e \end{array}\right.$$

It can be seen that e simultaneously contains the outputs of both branches and affects the partial derivatives of both. This mechanism enforces joint optimization, reducing divergence between adjacent lead representations and promoting wall-wise synergy. Consequently, localized spatial information is integrated into a unified wall-level embedding.

In summary, prior frameworks use either individual projections or unconstrained global configurations, whereas WAVE uses a shared projection. The shared projection allows features from different branches to interact during training, enabling collaborative optimization and thereby capturing the wall-wise synergy. Instead of treating multi-lead ECG signals as generic multi-channel time-series data, WAVE explicitly maps the spatial-anatomical properties of the heart into a structural network bias. It transforms the physical lead-to-wall correspondence into an algorithmic regularization mechanism, establishing a domain-specific representation scheme. This scheme models how regional cardiac dynamics correlate across different physiological perspectives of the heart.

### 3.3. Core Mechanisms of WAVE

Given an ECG $X\in R^{C\times L}$, its two views $\left(X_{1},X_{2}\right)$ are obtained through transformations $T_{1}$ and $T_{2}$: $X_{1}=T_{1}\left(X\right),X_{2}=T_{2}\left(X\right)$. *C* and *L* are the number of leads and the length of the ECG, respectively. These transformations originate from RRC-TO, as it has been demonstrated to be both simple and effective [8]. Afterwards, $X_{1}$ and $X_{2}$ are split according to the leads to obtain the corresponding lead-wise views, as follows:

(4) $$\left\{\begin{array}{c} \left\{X_{1}^{\mathrm{II}},X_{1}^{\mathrm{III}},X_{1}^{V1},\ldots ,X_{1}^{V6}\right\}=\mathrm{split}\left(X_{1}\right)\\ \left\{X_{2}^{\mathrm{II}},X_{2}^{\mathrm{III}},X_{2}^{V1},\ldots ,X_{2}^{V6}\right\}=\mathrm{split}\left(X_{2}\right) \end{array}\right.,X_{i}^{\mathrm{II}},\ldots ,X_{i}^{V6}\in R^{1\times L},i=1,2$$

As an encoder, MBN processes the views to obtain feature vectors. Inspired by BYOL, the encoder can be categorized into an online encoder and a target encoder. They process the views as:

(5) $$\left\{\begin{array}{c} h_{1}^{ld}=f_{\theta }^{ld}\left(X_{1}^{ld}\right)\\ h_{2}^{ld}=f_{\xi }^{ld}\left(X_{2}^{ld}\right) \end{array},ld\in \left\{\mathrm{II},\mathrm{III},\mathrm{V1},\ldots ,\mathrm{V6}\right\}\right.$$

where $h_{1}^{ld}$ and $h_{2}^{ld}$ are the feature vectors of a lead, $f_{\theta }^{ld}$ and $f_{\xi }^{ld}$ are the online and target encoders corresponding to the lead, respectively. θ and ξ denote the parameters of the encoders. According to the correspondence between leads and cardiac walls (as shown in Figure 1), the projectors further process the feature vectors of leads corresponding to the same wall to create the wall embeddings:

(6) $$\left\{\begin{array}{c} z_{\theta }^{\mathrm{IN}}=g_{\theta }^{\mathrm{IN}}\left(\mathrm{concat}\left(h_{1}^{\mathrm{II}},h_{1}^{\mathrm{III}}\right)\right),z_{\xi }^{\mathrm{IN}}=g_{\xi }^{\mathrm{IN}}\left(\mathrm{concat}\left(h_{2}^{\mathrm{II}},h_{2}^{\mathrm{III}}\right)\right)\\ z_{\theta }^{\mathrm{SE}}=g_{\theta }^{\mathrm{SE}}\left(\mathrm{concat}\left(h_{1}^{V1},h_{1}^{V2}\right)\right),z_{\xi }^{\mathrm{SE}}=g_{\xi }^{\mathrm{SE}}\left(\mathrm{concat}\left(h_{2}^{V1},h_{2}^{V2}\right)\right)\\ z_{\theta }^{\mathrm{AN}}=g_{\theta }^{\mathrm{AN}}\left(\mathrm{concat}\left(h_{1}^{V3},h_{1}^{V4}\right)\right),z_{\xi }^{\mathrm{AN}}=g_{\xi }^{\mathrm{AN}}\left(\mathrm{concat}\left(h_{2}^{V3},h_{2}^{V4}\right)\right)\\ z_{\theta }^{\mathrm{LA}}=g_{\theta }^{\mathrm{LA}}\left(\mathrm{concat}\left(h_{1}^{V5},h_{1}^{V6}\right)\right),z_{\xi }^{\mathrm{LA}}=g_{\xi }^{\mathrm{LA}}\left(\mathrm{concat}\left(h_{2}^{V5},h_{2}^{V6}\right)\right) \end{array}\right.$$

where “IN”, “SE”, “AN”, and “LA” denote the inferior, septal, anterior, and lateral walls, respectively. Given that wl∈{IN,SE,AN,LA}, the online projector $g_{\theta }^{wl}$ produces an online embedding, while the target projector $g_{\xi }^{wl}$ produces a target embedding. Following the practice of BYOL, the predictor $q_{\theta }^{wl}$ is applied to the online embedding to yield a prediction $p_{\theta }^{wl}$ as follows:

(7) $$\left\{\begin{array}{c} p_{\theta }^{\mathrm{IN}}=q_{\theta }^{\mathrm{IN}}\left(z_{\theta }^{\mathrm{IN}}\right),p_{\theta }^{\mathrm{SE}}=q_{\theta }^{\mathrm{SE}}\left(z_{\theta }^{\mathrm{SE}}\right)\\ p_{\theta }^{\mathrm{AN}}=q_{\theta }^{\mathrm{AN}}\left(z_{\theta }^{\mathrm{AN}}\right),p_{\theta }^{\mathrm{LA}}=q_{\theta }^{\mathrm{LA}}\left(z_{\theta }^{\mathrm{LA}}\right) \end{array}\right.$$

For pretraining, the alignment task is performed using the online predictions and target projections. First, features of the same cardiac wall across different views are aligned to promote invariance to transformations. In other words, the intra-wall correlation should be explored for each cardiac wall. The MSE-based loss is employed to measure this invariance/correlation, as follows:

(8) $$\begin{array}{cc} L_{\mathrm{inv}} & =\sum_{wl\in M}{∥{\bar{p}}_{\theta }^{wl}-{\bar{z}}_{\xi }^{wl}∥}_{2}^{2},M=\left\{\mathrm{IN},\;\mathrm{SE},\;\mathrm{AN},\;\mathrm{LA}\right\}\\ {\bar{p}}_{\theta }^{wl} & =\frac{p_{\theta }^{wl}}{{∥p_{\theta }^{wl}∥}_{2}},{\bar{z}}_{\xi }^{wl}=\frac{z_{\xi }^{wl}}{{∥z_{\xi }^{wl}∥}_{2}} \end{array}$$

where ${∥\cdot ∥}_{2}$ is to compute the L2 norm. wl∈{IN,SE,AN,LA} denotes the cardiac wall corresponding to the embedding/prediction. Minimizing this loss can enhance the intra-wall correlation, making the features invariant to transformations. Second, the alignment task explores the inter-wall correlation. Given an online prediction $p_{\theta }^{wl}$, the loss is defined to minimize its distance to the mean target embedding of other walls, as follows:

(9) $$\begin{array}{cc} L_{\mathrm{int}} & =\sum_{wl\in M}{∥{\bar{p}}_{\theta }^{wl}-{\bar{z}}_{\xi }^{\left(M\setminus wl\right)}∥}_{2}^{2},M=\left\{\mathrm{IN},\;\mathrm{SE},\;\mathrm{AN},\;\mathrm{LA}\right\}\\ {\bar{p}}_{\theta }^{wl} & =\frac{p_{\theta }^{wl}}{{∥p_{\theta }^{wl}∥}_{2}},{\bar{z}}_{\xi }^{\left(M\setminus wl\right)}=\frac{z_{\xi }^{\left(M\setminus wl\right)}}{{∥z_{\xi }^{\left(M\setminus wl\right)}∥}_{2}},z_{\xi }^{\left(M\setminus wl\right)}=\frac{\sum_{wl\in (M\setminus wl)}z_{\xi }^{wl}}{3} \end{array}$$

where (M∖wl) denotes all walls except wl. Although the inter-wall alignment task encourages each wall’s prediction to match the mean embedding of the remaining walls, lead-wise diversity is preserved through two constraints. First, WAVE employs an MBN with dedicated 1D CNN encoders. These encoders extract lead-specific features, preserving inherent inter-lead and inter-wall variability before projection. Second, Equation (9) excludes each wall’s own representation from its alignment target (M∖wl). This prevents trivial collapse to a global constant. As a result, the model learns a coordinated yet differentiated multi-view representation of cardiac activity. The two losses above constitute the final pretraining loss $L_{\mathrm{pt}}$:

(10) $$L_{\mathrm{pt}}=L_{\mathrm{inv}}+L_{\mathrm{int}}$$

The parameters θ are optimized by minimizing the loss $L_{\mathrm{pt}}$, while ξ is updated as the exponential moving average (EMA) of θ:

(11) $$\left\{\begin{array}{c} \theta \leftarrow {\arg\min}_{\theta }L_{\mathrm{pt}}\\ \xi \leftarrow \tau \cdot \xi +(1-\tau )\cdot \theta \end{array}\right.$$

where τ∈[0,1] is the target decay rate. In other words, the pretraining loss $L_{\mathrm{pt}}$ backpropagates error signals exclusively to update the online network parameters θ. A stop-gradient operation is applied to the target network to shield its parameters (ξ) from backpropagation. These parameters are updated solely via the smooth EMA trajectory of the online parameters. Inspired by BYOL [26,35], WAVE prevents representation collapse by leveraging an asymmetric joint-embedding architecture. This architecture is governed by three decoupled mechanisms. The first is the online predictor, which breaks the structural symmetry between the online and target pipelines. The second is the stop-gradient boundary, which isolates target representations from immediate gradient-driven updates. The third is the slow-moving momentum schedule of the target parameters. Overall, these elements prevent the system from converging to trivial constant representations.

**Figure 2:**
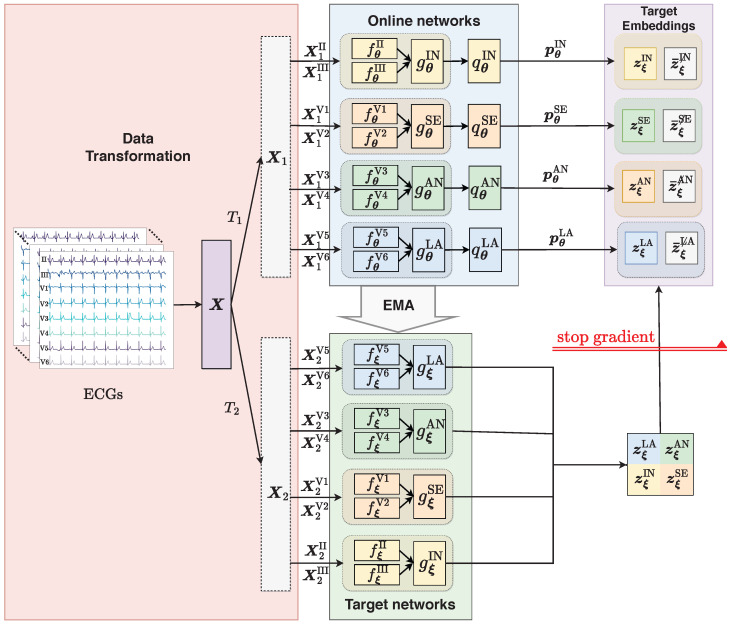
The pretraining mechanism of WAVE. 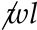, wl∈{IN,SE,AN,LA} denotes all walls except wl. Original ECGs are transformed into two views and fed into the online and target networks. Loss is computed between online predictions and target embeddings to update the online network. The target network is updated via EMA from the online network.

After pretraining, only the online encoders are retained for downstream tasks, while all other components are discarded. Figure 2 illustrates the pretraining mechanism of WAVE. In downstream tasks, the outputs of the pretrained encoders for different leads are concatenated to form a global feature, which is then fed into a linear classifier for classification:

(12) $$\hat{y}=\sigma \left(\omega \cdot \mathrm{concat}\left(h^{\mathrm{II}},h^{\mathrm{III}},h^{V1},\ldots ,h^{V6}\right)+b\right)$$

where σ(·) denotes the softmax function, ω and b are the weight and bias of the linear classifier, respectively. $\hat{y}$ is the final classification result. In addition, the cross-entropy loss is employed to guide training. The quality of pretraining determines the model’s performance on downstream tasks.

## 4. Results

### 4.1. Datasets

This study employs four ECG databases from the PhysioNet Challenge 2021 [36], including the Ningbo First Hospital (NFH) database, Physikalisch-Technische Bundesanstalt (PTB)-XL database, China Physiological Signal Challenge (CPSC) 2018 database, and Chapman database. Among them, the NFH database is used for pretraining, while the other three databases are used for downstream tasks (i.e., ECG classification). The PTB-XL database contains five categories: normal (NORM), myocardial infarction (MI), ST-T change (STTC), hypertrophy (HYP), and conduction disturbance (CD). The CPSC database contains nine categories: NORM, atrial fibrillation (AF), first-degree atrioventricular block (I-AVB), left bundle branch block (LBBB), right bundle branch block (RBBB), premature ventricular contraction (PVC), premature atrial contraction (PAC), ST-segment depression (STD), and ST-segment elevation (STE). The Chapman database includes four categories: AF, general supraventricular tachycardia (GSVT), sinus bradycardia (SB), and sinus rhythm (SR). Except for the CPSC database, the ECG recordings in the other datasets have a duration of 10 s. The dataset construction protocols are as follows:

- NFH: The original ECG records are 10 s in length with a sampling rate of 500 Hz (yielding 5000 sampling points per lead). These signals are resampled to a uniform length of 2048 points. As this database is utilized for self-supervised pretraining, no labels or train-validation-test splits are required.
- PTB-XL: The original records are 10 s long at 500 Hz (5000 points) and are similarly resampled to 2048 points. To partition the dataset, the data are divided into training, validation, and test sets. This follows the standardized patient-level split strategy recommended in the original PTB-XL publication [37].
- CPSC: The original ECG records vary in length. Protocols from prior literature [11,12,13] are followed to either crop or zero-pad the signals to exactly 10 s. The dataset is partitioned into training, validation, and test sets using a 7:1:2 patient-level split ratio. This is consistent with the strategy utilized in [11,12,13].
- Chapman: The original ECG signals have a duration of 10 s with a sampling rate of 500 Hz (5000 points) and are resampled to 2048 points. Similar to the CPSC database, a 7:1:2 patient-level split ratio is applied for the training, validation, and test sets, as adopted in previous studies [11,12,13].

Moreover, samples with multiple labels are removed. Following all the aforementioned filtering and processing steps, the detailed class distributions for each dataset are provided in Table 1.

### 4.2. Evaluation Paradigm and Metrics

This work adopts two commonly used SSL evaluation protocols, namely linear probing and fine-tuning, to assess model performance. The downstream model consists of a pretrained encoder followed by a linear classifier. During downstream training, linear probing keeps the encoder frozen, while fine-tuning updates it. The checkpoint achieving the highest performance on the validation set is selected. Its final generalization is evaluated on the independent test set. To further examine the model’s capability under limited labeled ECG data, fine-tuning is conducted using only 10% of the training set.

The evaluation metrics are area under the receiver operating characteristic curve (AUROC) and area under the precision–recall curve (AUPRC). Compared with accuracy and F1-score, AUROC and AUPRC consider model performance across various classification thresholds. Thus, they provide a more comprehensive assessment of generalization ability.

### 4.3. Architecture and Parameter Settings

**Figure 3:**
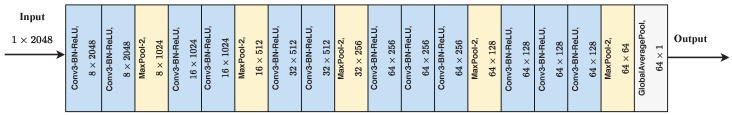
Architecture of the single-lead CNN encoder. **Conv3-BN-ReLU**: a block consisting of a 1D convolutional layer (kernel size = 3), batch normalization, and ReLU activation. **MaxPool-2**: a 1D max-pooling layer with a stride of 2. **GlobalAveragePool**: a global average pooling layer. The multiplied numbers represent the output feature map dimensions of each layer (number of channels × length).

**Figure 4:**
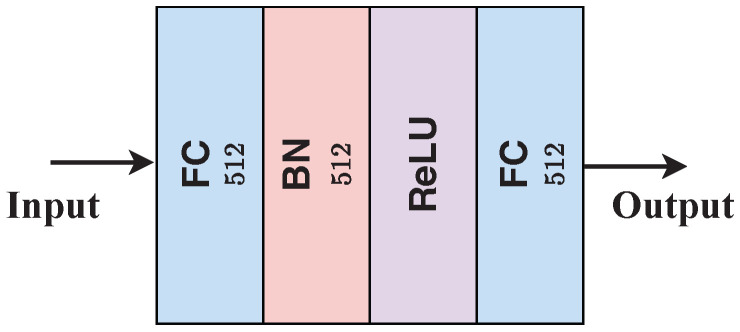
Architecture of the projector/predictor. **FC**: a fully-connected layer; **BN**: batch normalization; **ReLU**: ReLU activation. The numbers represent the output feature dimensions of each layer.

WAVE adopts the same MBN encoder architecture as proposed in [12,13]. It consists of eight branches corresponding to eight ECG leads, where each branch is a VGGNet-style 1D CNN, as shown in Figure 3. Each branch outputs a 64-dimensional feature vector. As presented in Figure 4, both the projector and predictor use a two-layer multi-layer perceptron. A batch normalization layer and a ReLU activation function are placed between the two fully-connected layers. The hidden dimension and the embedding dimension are both set to 512.

Both pretraining and downstream training use the Adam optimizer (exponential decay rates $\beta_{1}=0.9,\beta_{2}=0.999$). The learning rate, batch size, and number of epochs for pretraining are set to 0.001, 128, and 100, respectively. The target decay τ is 0.996. The RRC-TO augmentation parameters are configured with a random resize length of 1024∼2048 and a time-out mask length of 0∼1024. For linear probing, the learning rate, batch size, and epoch count remain 0.001, 128, and 100. For fine-tuning, the learning rate is changed to 0.0001, while the other parameters are kept unchanged. All models are implemented using PyTorch v1.11 and trained on an Nvidia RTX 2080Ti GPU.

### 4.4. Baselines

To validate the superiority of WAVE, the following SSL methods are selected as baselines for performance comparison: SimCLR [3], MoCo [4], BYOL [26], SimSiam [27], BT [28], VICReg [29], DLC [11], BELL [12], and LCD [13]. All baselines are reproduced utilizing their official open-source codebases or strictly following the algorithmic frameworks from their original literature. They share the same encoder architecture, data transformations, and general training configurations (i.e., optimizer type, base learning rate, batch size, and the number of pretraining/downstream epochs). Notably, utilizing the same encoder architecture, data transformations, and general training configurations ensures fair comparison of training budgets across all methods.

### 4.5. Overall Performance

**Figure 5:**
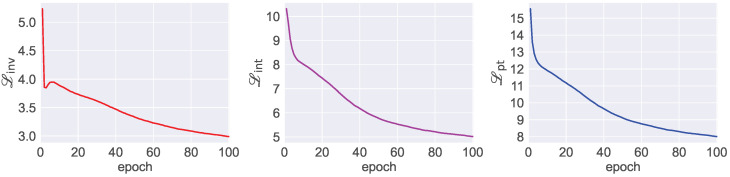
Changes in the losses during pretraining. The values represent the average loss over each epoch.

The model is first pretrained on the NFH dataset, and the loss curves are shown in Figure 5. It can be observed that both $L_{\mathrm{inv}}$ and $L_{\mathrm{int}}$ decrease significantly and converge as pretraining progresses. This indicates that both objectives effectively contribute to the pretraining process rather than a single loss dominating the optimization. After pretraining, all online encoders are transferred to the PTB-XL, CPSC, and Chapman databases for downstream training and evaluation. Depending on the evaluation paradigm (linear probing or fine-tuning), the corresponding experimental results are reported below. The average results are obtained from multiple independent experimental runs under ten different random seeds. They are presented in the format of mean ± standard deviation.

#### 4.5.1. Linear Probing Performance

Table 2 presents the performance of WAVE and the baselines under the linear probing paradigm. Compared with general-purpose SSL methods such as SimCLR and MoCo, WAVE exhibits a significant performance advantage. Considering the best-performing general-purpose method, BYOL, WAVE achieves improvements of 1.39–12.34% across different metrics, highlighting the benefit of incorporating ECG-specific mechanisms. Compared with ECG-specific SSL methods such as DLC, BELL, and LCD, WAVE still outperforms them. In particular, the maximum performance gain reaches 3.14% when compared with the best baseline for each task. This improvement is observed in PTB-XL classification measured by AUPRC (0.6715 vs. 0.7029). The results demonstrate that the design principles of WAVE effectively enable the extraction of high-quality ECG representations. In summary, WAVE outperforms all baselines under the linear probing paradigm, validating its effectiveness. Note that the three databases cover diverse clinical categories and types of arrhythmia. The consistent superiority of WAVE across these diagnostic tasks provides empirical evidence of its robust adaptability to different ECG interpretation contexts.

#### 4.5.2. Fine-Tuning Performance

**Figure 6:**
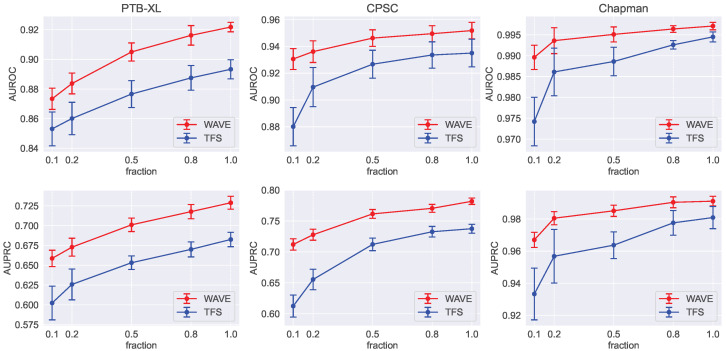
Performance of models initialized with WAVE pretraining versus models trained from scratch (TFS), under different fractions of labeled training data (10%, 20%, 50%, 80%, and 100%). Error bars represent the standard deviation across multiple runs.

Table 3 presents the results of WAVE and other baselines under the fine-tuning setting. Overall, WAVE consistently outperforms all baselines. Notably, only 10% of the labeled training data is used for fine-tuning. WAVE still achieves the best performance, demonstrating its ability to reduce reliance on labeled data. In addition, a comparison is conducted between a model initialized with WAVE pretraining and a model trained from scratch. This is evaluated under different proportions of labeled training data (10%, 20%, 50%, 80%, and 100%). The test results are shown in Figure 6. It is observed that the pretrained model consistently maintains a clear advantage. Even when the full training set (100%) is used, pretraining still provides noticeable performance gains. In summary, WAVE enables the model to learn rich ECG prior knowledge during pretraining. As a result, strong performance can be achieved with limited labeled data, substantially reducing reliance on manual annotation.

### 4.6. Ablation Analysis

This subsection investigates the impact of the key modules and operations in WAVE on model performance. Notably, all experiments are conducted under the linear probing paradigm.

#### 4.6.1. The Combined Loss

The pretraining loss $L_{\mathrm{pt}}$ is composed of two losses, $L_{\mathrm{inv}}$ and $L_{\mathrm{int}}$. As shown in Section 4.5, both $L_{\mathrm{inv}}$ and $L_{\mathrm{int}}$ contribute to guiding the pretraining process. However, whether they both benefit downstream performance remains to be verified. To this end, experiments are conducted where only $L_{\mathrm{inv}}$ or only $L_{\mathrm{int}}$ is used during pretraining. The results are presented in Table 4. Both $L_{\mathrm{inv}}$ and $L_{\mathrm{int}}$ alone yield inferior performance compared to the combined $L_{\mathrm{pt}}$. This indicates that combining the two losses is necessary. It improves pretraining quality and enhances downstream performance.

#### 4.6.2. Shared Projection

**Figure 7:**
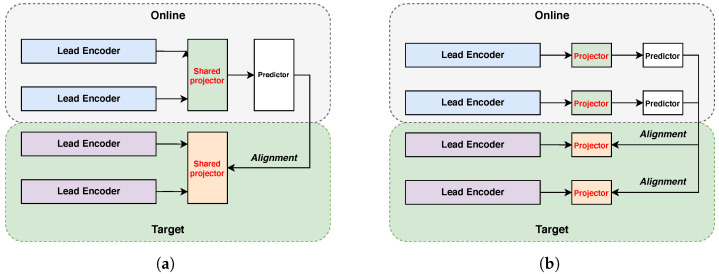
Architectures of the alignment task during pretraining: (**a**) using the shared projection to generate embeddings; (**b**) using the independent projection to generate embeddings for each lead.

The dual alignment in WAVE is performed on embeddings generated by shared projection, as illustrated in Figure 7a. The qualitative analysis in Section 3.2 indicates that the shared projection helps capture wall-wise synergy. However, its performance gain still requires experimental verification. To this end, an alternative setting is evaluated: the alignment task is performed on embeddings produced by independent projectors to pretrain all lead encoders, as shown in Figure 7b. Following the same principle, the loss function is defined as the MSE between lead embeddings. The results are reported in Table 5. The shared projection outperforms the independent projection. It achieves up to a 3.41% improvement (PTB-XL classification performance measured by AUPRC: 0.6688 vs. 0.7029).

#### 4.6.3. Wall-Based Concatenation

WAVE concatenates the features of leads corresponding to the same cardiac wall and feeds them into the shared projection to obtain embeddings. It is essential to verify the rationality of this concatenation, i.e., to confirm whether it indeed benefits downstream task performance. To this end, the eight lead features are randomly and uniformly partitioned into four groups, with two leads in each group. The features of leads within the same group are concatenated and fed into the shared projection to generate embeddings. Pretraining is performed with the above dual-alignment task. The encoders are then transferred to downstream tasks. The experiment is repeated ten times, and the averaged downstream performance is reported in Table 6. It can be observed that the wall-based concatenation still maintains a certain performance advantage, confirming its necessity. Conversely, these results suggest that alternative lead-wall mappings affect the method’s efficacy. Arbitrary configurations generally underperform compared to our clinically grounded design.

To assess the statistical significance of the performance difference between random grouping and the proposed wall-based grouping, the median-performing model across 10 trials is selected. An empirical bootstrap analysis with 1000 resamples is then conducted. *p*-values are computed for both AUROC and AUPRC. The results indicate that wall-based grouping yields a statistically significant improvement (*p* < 0.05) over random grouping on both the PTB-XL and CPSC datasets. In contrast, no significant difference is observed on the Chapman dataset (*p* > 0.05). This discrepancy may be attributed to two factors. First, Chapman is a relatively easier task. Both methods already achieve very high performance (AUROC and AUPRC typically exceed 0.97), leaving limited room for statistically detectable gains. Second, Chapman focuses on cardiac rhythm classification. Abnormalities are globally distributed across leads rather than localized to anatomically specific regions. As a result, the anatomical wall-based prior provides limited additional benefit compared to tasks involving localized morphological patterns such as MI. To summarize, the anatomical wall-based prior provides a clear advantage. Nevertheless, its effectiveness depends on the difficulty and physiological characteristics of the task.

#### 4.6.4. Mean Target Embedding

According to Equation (9), when computing the inter-wall alignment loss $L_{\mathrm{int}}$, the target embeddings of the other walls are averaged and used as the target for fitting the online embedding. However, an alternative formulation exists. The loss can be computed between each wall’s online embedding and the target embeddings of every other wall separately. These losses are then summed to form the overall objective:

(13) $$L_{\mathrm{int}}^{\prime }=\sum_{wl}L_{\mathrm{int}}^{wl}=\sum_{wl}\sum_{wl^{\prime }\neq wl}{∥{\bar{p}}_{\theta }^{wl}-{\bar{z}}_{\xi }^{wl^{\prime }}∥}_{2}^{2},M=\left\{\mathrm{IN},\;\mathrm{SE},\;\mathrm{AN},\;\mathrm{LA}\right\},wl,wl^{\prime }\in M$$

This loss is used to replace $L_{\mathrm{int}}$ in Equation (9) for pretraining and downstream evaluation. The results are shown in Table 7. It can be observed that mean target embedding achieves better performance. This is because computing losses with respect to different targets ($L_{\mathrm{int}}^{wl},wl\in \left\{\mathrm{IN},\;\mathrm{SE},\;\mathrm{AN},\;\mathrm{LA}\right\}$) may lead to gradient conflicts, which hinder model optimization. In contrast, mean target embedding defines a single target that mitigates localized noise and reduces conflicting gradients, leading to more stable pretraining.

Furthermore, the inter-wall alignment objective operates only in a projected latent space (via the projector and predictor). It does not operate directly on structural representations. It serves as a soft regularizer that captures shared cardiac rhythm and patient-level semantics, without enforcing hard structural uniformity. This helps preserve wall-specific physiological information in the backbone encoder. During downstream evaluation, the pretrained model is further optimized with task-specific supervision. The optimization reactivates and strengthens wall-specific discriminative features relevant to target pathologies. Moreover, consistent performance gains across multiple downstream tasks provide indirect evidence of high-quality representations, indicating that wall-specific information is well preserved during pretraining.

## 5. Discussion

### 5.1. Qualitative Analysis and Visualization

**Figure 8:**
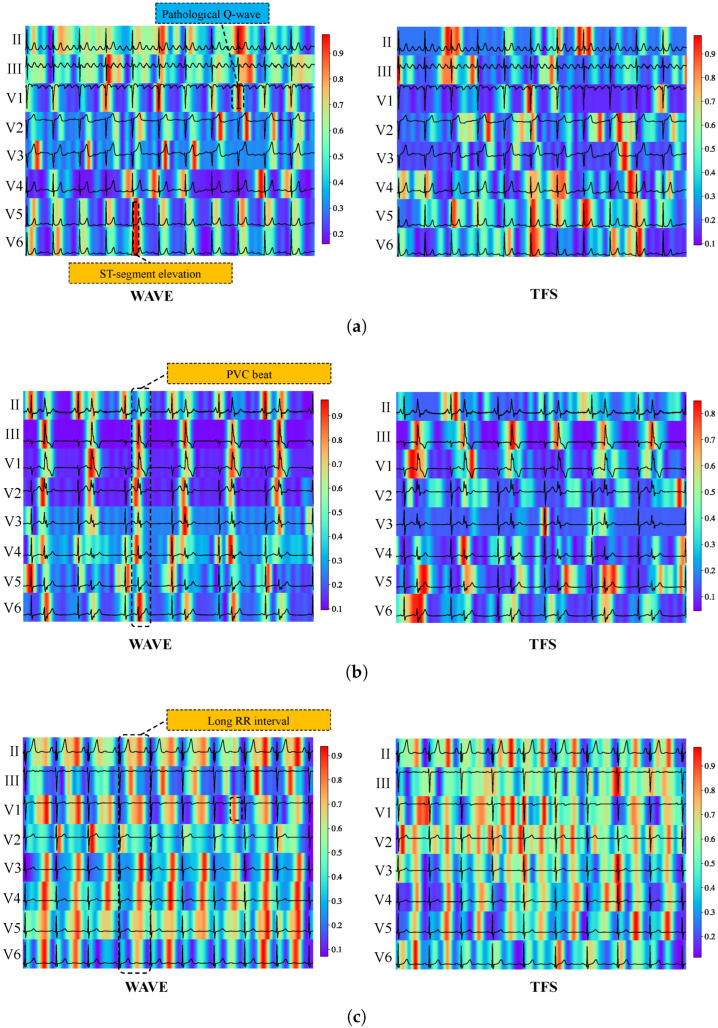
Grad-CAM visualizations of models trained with 10% labeled data under fine-tuning. (**a**) An MI sample from the PTB-XL dataset. (**b**) A PVC sample from the CPSC dataset. (**c**) An SB sample from the Chapman dataset. Each example compares the activation maps of the pretrained model (WAVE) and the model trained from scratch (TFS).

According to the fine-tuning results in Section 4.5.2, the model pretrained with WAVE achieves strong ECG classification performance even when only limited labeled data are available. The results indicate a reduced dependency on labels. In theory, this can only be achieved if pretraining enables the model to better capture key ECG patterns. Further experimental verification is still required. Therefore, gradient-weighted class activation mapping (Grad-CAM) is applied to visualize the model trained with only 10% of the downstream labels. The pretrained model is compared against the model trained from scratch to highlight the effect of pretraining. Several representative examples are shown in Figure 8. Figure 8a presents an MI sample from the PTB-XL database. The pretrained model can capture more typical MI-related patterns, such as pathological Q waves in lead V1 and ST-segment elevation in leads V5 and V6. However, the model trained from scratch only captures a subset of these patterns. Figure 8b shows a PVC sample from the CPSC database. The pretrained model successfully detects most PVC beats, while the model trained from scratch again captures only part of them. Figure 8c shows an SB sample from the Chapman database. SB samples are characterized by abnormally low heart rate, resulting in prolonged RR intervals. The pretrained model focuses on the prolonged RR intervals and exhibits a clear rhythm-aware activation pattern. The model trained from scratch also detects long RR intervals but produces a more scattered activation distribution, indicating less accurate rhythm perception. In summary, WAVE pretraining enables the model to capture richer and more informative ECG features. Such performance gains hold even when labeled data are extremely limited.

**Figure 9:**
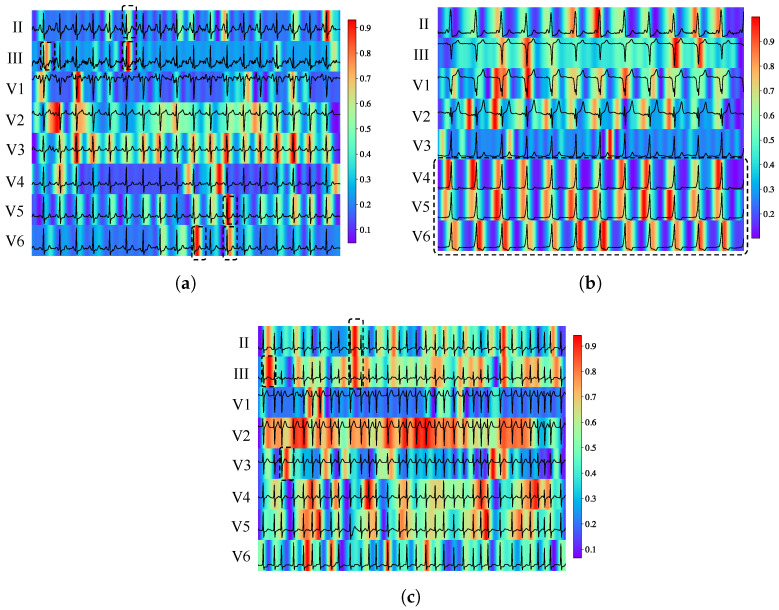
Grad-CAM visualizations of representative failure cases. (**a**) A NORM sample misclassified as STTC due to mild ST-segment depression, highlighted by black dashed boxes. (**b**) An RBBB sample misclassified as PVC due to wide QRS complexes and inverted T waves (V4∼V6). (**c**) An AF sample misclassified as GSVT due to co-existing severe tachycardia and irregular RR intervals (e.g., intervals highlighted in leads II and III).

Additionally, representative failure cases from downstream tasks are analyzed via Grad-CAM visualizations, as shown in Figure 9. Figure 9a shows a sample with a ground-truth label of NORM that is misclassified as STTC. As indicated in the activation map, the model focuses on mild ST-segment depression in leads II, III, V5, and V6. These morphological variations are subtle and insufficient for an official clinical diagnosis of STTC. However, they trigger the model’s sensitivity to ST-T abnormalities, leading to the misclassification. Figure 9 presents a sample with a ground-truth label of RBBB that is misclassified as PVC. The activation map reveals that the model primarily concentrates on the wide QRS complexes and inverted T waves in leads V4 to V6. These morphological features share substantial similarities with PVC. This overlap may confound the classification decision. Figure 9c shows a sample with a ground-truth label of AF that is misclassified as GSVT. The model successfully detects irregular RR intervals in leads II, III, and V3, which represents a key pathological pattern of AF. However, the record also exhibits an extremely elevated ventricular rate of approximately 198 beats per minute. This co-occurrence of severe tachycardia and rhythm irregularity obscures the distinction between the two classes, resulting in the misclassification. In summary, the analysis of these failure cases shows that the model still focuses on clinically relevant pathological patterns even in misclassified cases. The primary struggles occur under conditions of cross-class feature similarities, co-existing diagnostic patterns, or subtle morphological variations. These factors represent fundamental challenges in automated computer-aided diagnosis.

**Figure 10:**
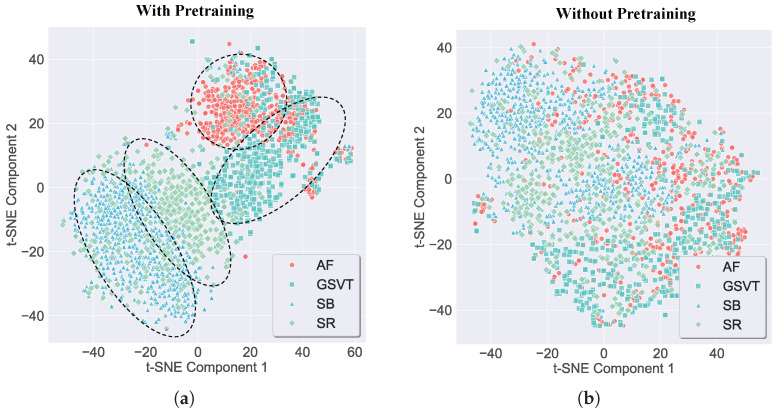
t-SNE visualization of feature representations on the Chapman test set. (**a**) pretrained encoder; (**b**) encoder without pretraining.

The above Grad-CAM results focus on explaining local ECG patterns, whereas interpreting the global separability of the pretrained ECG features is also important. To this end, the features output by the pretrained encoder are visualized using t-distributed stochastic neighbor embedding (t-SNE) to examine their global separability. However, the model does not achieve particularly high absolute performance on the PTB-XL and CPSC datasets (especially in terms of AUPRC). In addition, the number of classes is relatively large. As a result, generating a clear t-SNE visualization may be difficult. Therefore, only the Chapman database is used here for t-SNE visualization of the pretrained encoder features. These features are further compared with those produced by an encoder without pretraining (i.e., randomly initialized). It should be noted that neither of the two encoders is fine-tuned for downstream tasks. Figure 10 shows the t-SNE scatter plots of the feature representations on the Chapman test set. Even without fine-tuning, the features produced by the pretrained encoder exhibit better separability. In contrast, the features from the encoder without pretraining show a high degree of inter-class overlap and poor separability. In summary, WAVE pretraining enables the model to learn more discriminative features, which lays the foundation for its strong performance on downstream tasks.

### 5.2. Limitations and Future Works

Although WAVE has demonstrated strong advantages in both theory and experiments, several limitations remain to be addressed:

- The current validation of WAVE is strictly limited to ECG classification tasks. Broader clinical validation across other ECG applications, such as denoising, waveform segmentation, or heart rate estimation, is still lacking.
- Its deployment scenarios remain constrained. This work primarily focuses on eight-lead ECGs. Real-world clinical monitoring, especially wearable or single-lead scenarios, has not yet been empirically validated.
- The incorporation of clinical domain knowledge remains limited. The current design only considers the anatomical correspondence between leads and cardiac walls, leaving richer medical priors unexplored.

Therefore, future work will focus on the broader clinical validation of WAVE across diverse ECG tasks and its adaptation to low-lead or wearable-device settings. WAVE can theoretically be extended to various clinical tasks (e.g., via task-specific modules) or low-lead scenarios (e.g., via representation distillation from a multi-lead teacher). However, these adaptations differ fundamentally from our current classification tasks. Effectively extending WAVE to these scenarios while fully leveraging its physiological priors remains a challenging and critical direction for future research. In addition, more comprehensive medical knowledge will be integrated into the model architecture and pretraining objectives, aiming to enhance both the performance and clinical interpretability of WAVE.

## 6. Conclusions

This paper introduces WAVE, a novel SSL method for ECG classification. WAVE systematically addresses the diversity, synergy, and correlation of multi-lead ECG signals. It designs model architecture and pretraining tasks to exploit unlabeled ECG and reduce reliance on labeled data. Experimental results demonstrate that WAVE consistently outperforms all baseline models across multiple evaluation settings, highlighting its effectiveness. Ablation studies further confirm that each key component contributes positively to pretraining quality, including the combined loss, shared projection, wall-based concatenation, and mean target embedding. Despite these advantages, WAVE has limitations regarding task scope, application scenarios, and incorporation of medical knowledge. Future work will address these issues to extend WAVE’s applicability and enhance its utility in real-world clinical settings. 

## Figures and Tables

**Table 1:** Statistic Information of the Datasets.

Database	Pretrain			
NFH	34,905			
	Class	Train	Val	Test
PTB-XL	NORM	7254	916	913
	CD	2048	234	256
	HYP	1353	172	184
	MI	416	64	56
	STTC	1907	256	243
	Total	12,978	1642	1652
CPSC-2018	NORM	1213	197	365
	AF	1289	168	342
	I-AVB	889	101	251
	LBBB	243	33	56
	RBBB	1964	292	589
	PAC	864	130	274
	PVC	1084	146	308
	STD	1148	178	345
	STE	264	58	68
	Total	8958	1303	2598
Chapman	AF	1583	186	449
	GSVT	1639	189	472
	SB	2804	315	769
	SR	1625	161	436
	Total	7651	851	2126

**Table 2:** Linear Probing Performance of WAVE and Baselines.

Method	PTB-XL		CPSC		Chapman	
	AUROC	AUPRC	AUROC	AUPRC	AUROC	AUPRC
SimCLR [3]	0.8842 ± 0.0082	0.6481 ± 0.0098	0.8882 ± 0.0099	0.5922 ± 0.0143	0.9762 ± 0.0048	0.9304 ± 0.0055
MoCo [4]	0.8680 ± 0.0066	0.6243 ± 0.0059	0.8934 ± 0.0091	0.6046 ± 0.0106	0.9631 ± 0.0036	0.9007 ± 0.0049
BYOL [26]	0.8788 ± 0.0045	0.6544 ± 0.0064	0.8935 ± 0.0072	0.6204 ± 0.0097	0.9782 ± 0.0024	0.9283 ± 0.0037
SimSiam [27]	0.7602 ± 0.0088	0.4411 ± 0.0186	0.7635 ± 0.0098	0.3492 ± 0.0160	0.8923 ± 0.0102	0.7119 ± 0.0188
BT [28]	0.8679 ± 0.0062	0.6395 ± 0.0091	0.8524 ± 0.0082	0.5231 ± 0.0101	0.9432 ± 0.0057	0.8302 ± 0.0096
VICReg [29]	0.8672 ± 0.0115	0.6255 ± 0.0178	0.8385 ± 0.0123	0.4901 ± 0.0143	0.9496 ± 0.0053	0.8625 ± 0.0083
DLC [11]	0.8912 ± 0.0050	0.6643 ± 0.0083	0.9291 ± 0.0065	0.7186 ± 0.0102	0.9867 ± 0.0020	0.9618 ± 0.0035
BELL [12]	0.8881 ± 0.0069	0.6614 ± 0.0077	0.9256 ± 0.0069	0.7242 ± 0.0087	0.9891 ± 0.0016	0.9682 ± 0.0023
LCD [13]	0.9017 ± 0.0031	0.6715 ± 0.0042	0.9271 ± 0.0056	0.7024 ± 0.0133	0.9879 ± 0.0010	0.9633 ± 0.0022
WAVE	0.9118 ± 0.0034	0.7029 ± 0.0047	0.9391 ± 0.0055	0.7438 ± 0.0092	0.9921 ± 0.0013	0.9762 ± 0.0018

**Table 3:** Fine-tuning Performance of WAVE and Baselines.

Method	PTB-XL		CPSC		Chapman	
	AUROC	AUPRC	AUROC	AUPRC	AUROC	AUPRC
SimCLR [3]	0.8330 ± 0.0117	0.5935 ± 0.0193	0.8987 ± 0.0120	0.6210 ± 0.0202	0.9845 ± 0.0067	0.9472 ± 0.0081
MoCo [4]	0.8202 ± 0.0181	0.5703 ± 0.0214	0.8981 ± 0.0105	0.6272 ± 0.0275	0.9734 ± 0.0079	0.9201 ± 0.0090
BYOL [26]	0.8413 ± 0.0104	0.6011 ± 0.0128	0.9023 ± 0.0079	0.6371 ± 0.0159	0.9793 ± 0.0052	0.9319 ± 0.0061
SimSiam [27]	0.7933 ± 0.0132	0.5192 ± 0.0261	0.8604 ± 0.0168	0.5629 ± 0.0297	0.9582 ± 0.0116	0.9004 ± 0.0189
BT [28]	0.8156 ± 0.0094	0.5602 ± 0.0129	0.8882 ± 0.0121	0.6129 ± 0.0169	0.9728 ± 0.0059	0.9225 ± 0.0077
VICReg [29]	0.8321 ± 0.0102	0.5894 ± 0.0141	0.8777 ± 0.0080	0.5982 ± 0.0135	0.9756 ± 0.0064	0.9276 ± 0.0084
DLC [11]	0.8501 ± 0.0109	0.6078 ± 0.0116	0.9145 ± 0.0082	0.6707 ± 0.0122	0.9879 ± 0.0045	0.9670 ± 0.0056
BELL [12]	0.8520 ± 0.0082	0.6312 ± 0.0119	0.9130 ± 0.0076	0.6698 ± 0.0130	0.9820 ± 0.0048	0.9574 ± 0.0062
LCD [13]	0.8516 ± 0.0077	0.6130 ± 0.0098	0.9175 ± 0.0088	0.6775 ± 0.0097	0.9885 ± 0.0033	0.9612 ± 0.0041
WAVE	0.8734 ± 0.0072	0.6587 ± 0.0103	0.9306 ± 0.0078	0.7121 ± 0.0093	0.9896 ± 0.0029	0.9670 ± 0.0047

**Table 4:** Downstream Performance under Different Pretraining Loss Configurations.

	PTB-XL		CPSC		Chapman	
	AUROC	AUPRC	AUROC	AUPRC	AUROC	AUPRC
$L_{\mathrm{inv}}$	0.8786 ± 0.0039	0.6453 ± 0.0045	0.8828 ± 0.0061	0.5832 ± 0.0098	0.9718 ± 0.0012	0.9103 ± 0.0019
$L_{\mathrm{int}}$	0.8888 ± 0.0046	0.6632 ± 0.0058	0.9297 ± 0.0052	0.7087 ± 0.0104	0.9888 ± 0.0021	0.9666 ± 0.0027
$L_{\mathrm{inv}}+L_{\mathrm{int}}$	0.9118 ± 0.0034	0.7029 ± 0.0047	0.9391 ± 0.0055	0.7438 ± 0.0092	0.9921 ± 0.0013	0.9762 ± 0.0018

$L_{\mathrm{inv}}+L_{\mathrm{int}}=L_{\mathrm{pt}}$.

**Table 5:** Downstream Performance under Different Projection Configurations.

	PTB-XL		CPSC		Chapman	
	AUROC	AUPRC	AUROC	AUPRC	AUROC	AUPRC
Independent	0.8911 ± 0.0044	0.6688 ± 0.0051	0.9277 ± 0.0047	0.7173 ± 0.0104	0.9827 ± 0.0010	0.9618 ± 0.0022
Shared	0.9118 ± 0.0034	0.7029 ± 0.0047	0.9391 ± 0.0055	0.7438 ± 0.0092	0.9921 ± 0.0013	0.9762 ± 0.0018

**Table 6:** Downstream Performance under Different Concatenation Methods.

	PTB-XL		CPSC		Chapman	
	AUROC	AUPRC	AUROC	AUPRC	AUROC	AUPRC
Random	0.9051 ± 0.0069	0.6791 ± 0.0095	0.9369 ± 0.0077	0.7298 ± 0.0147	0.9908 ± 0.0076	0.9696 ± 0.0088
Wall-based	0.9118 ± 0.0034	0.7029 ± 0.0047	0.9391 ± 0.0055	0.7438 ± 0.0092	0.9921 ± 0.0013	0.9762 ± 0.0018

**Table 7:** Downstream Performance under Different Methods for Target Computing.

	PTB-XL		CPSC		Chapman	
	AUROC	AUPRC	AUROC	AUPRC	AUROC	AUPRC
Separate Target	0.8920 ± 0.0052	0.6655 ± 0.0086	0.9249 ± 0.0042	0.7136 ± 0.0101	0.9890 ± 0.0011	0.9635 ± 0.0033
Mean Target	0.9118 ± 0.0034	0.7029 ± 0.0047	0.9391 ± 0.0055	0.7438 ± 0.0092	0.9921 ± 0.0013	0.9762 ± 0.0018

## Data Availability

The NFH, PTB-XL, CPSC, and Chapman databases are publicly available at: https://www.kaggle.com/datasets/erarayamorenzomuten/ningbo-first-hospital-12lead-ecg-database (accessed on 15 June 2026); https://physionet.org/content/ptb-xl/1.0.3/ (accessed on 15 June 2026); http://2018.icbeb.org/Challenge.html (accessed on 15 June 2026); https://figshare.com/collections/ChapmanECG/4560497/2 (accessed on 15 June 2026).
